# The impact of incorrectly recorded parentage on inferred genotypes over multiple generations

**DOI:** 10.1186/s40575-019-0074-3

**Published:** 2019-07-19

**Authors:** T. W. Lewis

**Affiliations:** 1 The Kennel Club, Clarges Street, London, W1J 8AB England; 2 School of Veterinary Medicine and Science, The University of Nottingham, Sutton Bonington Campus, Sutton Bonington, Leicestershire, LE12 5RD England

**Keywords:** Disease mutations, Genetic testing, Disease carriers, Hereditary clear status

## Abstract

The absence of selective pressure against recessive deleterious mutations in the heterozygous state means that virtually every individual will carry several such mutations which have arisen over time. The inflation in frequency of a few of these mutations due to selective processes during domestication and breed formation have left modern domestic dog breeds with a high burden of genetic disease due to mutations at single genes. This has stimulated research into the causal mutations, and a consequential market in DNA tests, which enable breeders to distinguish heterozygotes from wild type homozygotes and determine pairings that will avoid producing diseased progeny. The genotypes of progeny of parents with known genotypes themselves may in some cases be definitively inferred. Importantly, two parents homozygous for non-disease causing alleles, will produce progeny with the same genotype, which may be assigned to the offspring (e.g. as ‘hereditary clear’) without the need for further testing.

However, the veracity of assigned genotypes is dependent on the parentage being recorded without error, which is not the case in most species. Simulations presented here demonstrate that a modest rate of false paternity can result in a notable proportion of ‘hereditary clear’ assignments being false when ‘hereditary clear’ status is assigned across a number of generations (error rates exceeding 5% after 6 generations with a disease causing mutation frequency of 0.2). Erroneous assignment of ‘hereditary clear’ genotypes risks the production of puppies with the very disease for which a DNA test is available allowing avoidance. In light of these findings and to reduce the risks of producing puppies destined to be affected by such diseases, the Kennel Club has determined to limit the assignment of ‘hereditary clear’ status of registered dogs to 2 generations, with effect from January 2022.

## Plain English summary

The processes of domestication and breed formation have left modern domestic dog breeds with a high level of disease caused by single gene recessive mutations. Because possession of a single copy of a recessive deleterious mutation causes no ill-effects, there is no natural selection against them, meaning that every individual likely carries several rare disease causing mutations which have arisen over time and been passed down over generations. If a particular mutation becomes common in a breed, for example due to use of popular sires, then the disease it causes becomes particularly prevalent in that population. Frequently DNA tests become available as a consequence of research to identify the causal mutations.

Whether a puppy will carry none, one, or two copies of a known mutation is predictable in some cases where information on the parental carrier status (as is provided by a DNA test) is known. This enables breeders to make mating combinations to ensure no diseased progeny are produced. If two parents are clear for a mutation, then all their offspring will be clear too, and they can be assigned a ‘hereditary clear’ status, avoiding the need for repeat testing to confirm a known status.

However, the reliability of hereditary status rests on the documented parentage always being correct, which is not the case in most species, including dogs and humans. It is demonstrated in this study that, when ‘hereditary clear’ status is repeatedly assigned over a number of generations, a modest rate of false paternity can result in a notable proportion of ‘hereditary clear’ assignments being false, unless the disease causing mutation frequency is very low. These results demonstrate that incorrect recording of parentage can result in erroneous ‘hereditary clear’ status and risks the production of puppies with the very disease for which a DNA test is available to avoid. In light of these findings and to reduce the risks of producing puppies destined to be affected by such diseases, the Kennel Club has determined to limit the assignment of ‘hereditary clear’ status of registered dogs to 2 generations, with effect from January 2022.

## Background

Purebred dog breeds appear to be particularly predisposed to monogenic, autosomal recessively inherited disease. Dogs of modern domestic breeds have been shown to carry a higher number of deleterious mutations, and more commonly in duplicate (the homozygous state), than their wild progenitor the grey wolf, where they are more likely to occur only as a single copy (heterozygous state; [1]). Similarly, when comparing purebred and mixed breed dogs, Donner et al. [2] report that while mixed breed dogs were more likely to carry one or more of nine common disease causing mutations in the heterozygous state, purebred dogs were more likely to be homozygous for these mutations. It therefore appears that while there may be fewer actual deleterious mutations extant in individual purebred dog breed populations compared to mixed breed or wild populations, those that are present are so at higher frequencies and so more likely to manifest as diseased homozygotes.

It is extremely unlikely that any individual is entirely free of deleterious mutations (given the magnitude of the DNA molecule, the mutation rate, the likelihood of a disruptive versus advantageous outcome of a mutation on the resultant protein, and the high fidelity of DNA replication during gamete production). Nicholas [3] gives a theoretical example making a very conservative estimate of 4 in 100,000 humans being free of any lethal recessive mutations (which result in the death of the homozygote), and empirical data reveals that on average humans carry 250–300 recessive ‘loss of function’ mutations [4]. It seems reasonable to assume that these rates are similar in dogs, and that therefore virtually every individual carries some deleterious mutations. While fully-penetrant recessive deleterious mutations may cause catastrophic disease when in the homozygous state, when in the heterozygous state there is no selective disadvantage to the individual, and so natural selection against such mutations in the more common heterozygote state will not occur. The number of different deleterious mutations existing in the heterozygous state may therefore be sizeable in populations under natural selection [1], as is the case with humans. While deleterious alleles have been shown to exist in all canid populations, it is the processes of domestication and breed formation that have caused them to occur at a higher frequency in purebred populations, and so more commonly result in disease [1].

The objectives of domestication and breed formation are to produce individuals with particular characteristics, or traits, via genetic selection. In the case of domestication, selective sweeps on the canine genome suggest that the targets of selection were genes influencing brain function and behaviour [5]. Breed development has often been achieved via intense selection for particular traits in order to ‘fix type’ over a short number of generations, and several genes with large effects on traits such as body size, coat type, snout size and shape, and behaviour are reported as being swept to fixation in some breeds [6–9]. The selection objectives of both domestication and breed development are achieved via the same mechanisms: a small number of founder individuals, a largely closed breeding population and the extensive use of a small number of males as sires. The ‘over-use’ of some popular sires continues to this day, and has been determined as resulting in more widespread dissemination of mutant recessive alleles than line- or close-breeding [10]. Such selective mechanisms allow mutations, for which there is no selective disadvantage in the heterozygous state, to rise rapidly in frequency in populations, either hitchhiking within selective sweep regions centred on genes at which variants influence a trait of interest, or via random genetic drift, which is exacerbated through a small effective population size. Marsden et al. [1] demonstrate that it is historical selection during domestication and breed development, rather than more recently, that has left purebred dog populations with high frequencies of disease causing mutations.

Knowledge of genotype information relating to an autosomal locus with a ‘fully-penetrant’, recessively inherited, disease causing mutation, improves the accuracy of selection compared to just the use of phenotypic information, by enabling a selective disadvantage to be applied to the heterozygous state. Definitive identification among potential breeding candidates of the three possible genotypes, (i) homozygous for a non-disease causing (wild type) allele (often referred to as *clear*), (ii) homozygous for a disease-causing mutation (typically referred to as *affected*) prior to the development of clinical signs of disease, and (iii) heterozygotes (typically referred to as *carriers*), enables differentiation among outwardly identical, unaffected individuals. Breeders may therefore accurately identify which breeding candidates carry [a] disease causing mutant allele(s) and may pass it to their progeny. Since the rate that de novo mutation occurs is negligible (the average mammalian genome mutation rate is reported as being 2.2 × 10^− 9^ per base pair per year; [11]) the risk of a repeat mutation occurring (i.e. the exact same mutation occurring more than once in the exact same position) can safely be ignored, and the genotypes of progeny of particular parental genotype combinations are predictable. Thus, knowledge of genotypes improves the accuracy of selection against disease through the provision of the means to ensure that no affected individuals need inadvertently be produced from the breeding of outwardly healthy animals. Furthermore, selection against the disease causing mutation can rapidly reduce its frequency within the breed population.

The proliferation of DNA tests for Mendelian inherited disease in dogs has been rapid, with the current number of tests based on specific disease-associated mutations available globally likely to be in excess of 150 [2]. There is a strong uptake of genetic testing by dog breeders, as implied by the number of commercial test providers in the global market place [12], and both the virtual cessation in the production of affected puppies and general reduction in the frequency of disease causing mutations due to selection following public availability of DNA tests have been reported [13].

## Main text

### The concept of ‘hereditary clear’ status

In some cases, progeny genotypes may definitively be deduced from the parental genotypes; for example, in the case of an autosomal recessive disorder, all progeny of two ‘clear’ parents will also be ‘clear’. The Kennel Club, which records canine parentage (or pedigree) information together with results of health screening and DNA testing of registered dogs in the UK, has facilitated assignment of ‘hereditary clear’ (HC) status to such dogs. This has resulted in the propagation of HC status across generations for dogs whose breeders have invested in genetic testing and avoids the costs associated with testing every successive generation simply to confirm a known genotype.

However, as noted by Lewis and Mellersh [13], some dogs may erroneously be assigned HC status for a variety of reasons, for example failure of laboratory protocols, pedigree transcription error, or incorrectly identified matings leading to incorrectly recorded parentage. Left uncorrected, erroneous HC status may be passed from parent to progeny, likely remaining undetected for several generations, but risking the eventual production of affected animals from two breeding animals each with false HC status. Pedigree error is well documented in domesticated species, and has been estimated at a rate of between 1 and 9% in pedigree dog breeds [14]; this is high enough to make the erroneous assignment of HC status a very real possibility. Ostensibly, the probability of the occurrence of false HC status may be thought to directly equate to the rate of incorrect parentage, but this will be an over-estimate as it neglects the occurrence of clear homozygous genotypes in some progeny even when pedigree / parentage is false. In this paper, an effort is made to take account of this occurrence and the cumulative probability, or rate, of erroneous HC status is modelled over several generations at a given false-parentage rate and for a range of disease causing mutant allele frequencies. These theoretical results may be used as a guide to the risks in the assignment of HC status to dogs over a large number of generations.

### Model simulation

**Fig. 1:**
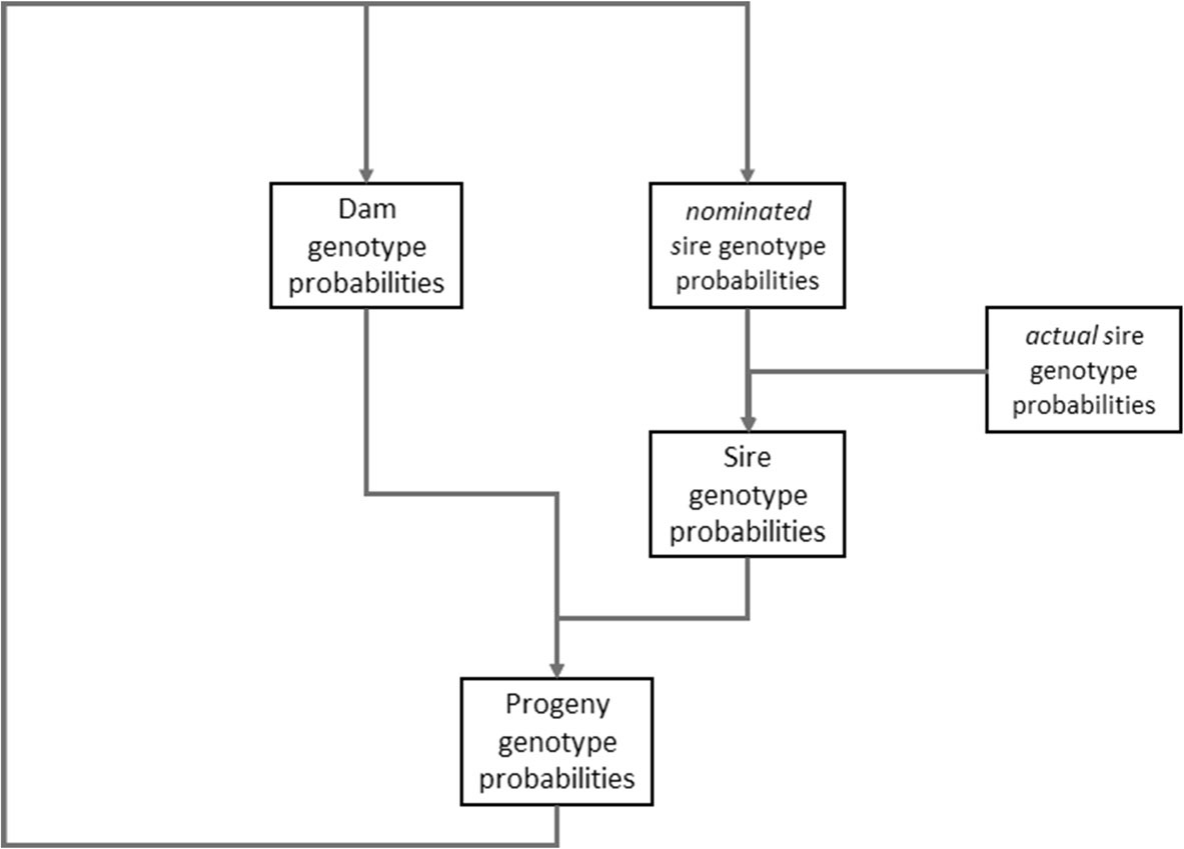
Diagrammatic representation of the iterative calculation of genotype probabilities of progeny over successive generations of individuals assigned ‘hereditary clear’ (HC) status. The actual sire genotype probabilities are the genotype frequencies in the background population assumed in HWE, occurring at a constant false paternity rate of 5%. The initial dam and nominated sire genotype probabilities are from individuals with genotypes known from testing, so P (AA) = 1. Thereafter over successive generations, the calculated genotype probabilities of progeny (assigned HC status) in generation n become the parent genotype probabilities in generation n + 1

The false hereditary clear rate (FHCR), or probability that the HC status assigned to an individual is incorrect, *n* generations after clear genotypes were determined in nominated ancestors via DNA testing, was modelled by calculating genotype probabilities at a hypothetical autosomal locus, ‘A’, with a recessive disease causing mutant allele (*a*) occurring at frequency *f(a)*, in a population assumed to start in Hardy-Weinberg equilibrium. The probability that parentage was incorrect was set to a constant figure of 0.05 (5%), but was considered for the sire only (the recording of the dam was presumed always to be correct), and so equates to a ‘false paternity’ rate. The genotype probabilities of parents and progeny over successive generations were calculated iteratively as illustrated in Fig. 1.

Genotype probabilities were calculated for: (i) nominated dams (assuming maternity was always correctly recorded), (ii) nominated sires at the rate that paternity was correctly recorded, and (iii) *actual* (rather than nominated) sires at the false paternity rate. Sire genotype probabilities were the sum of (ii) and (iii). Initial parental genotypes, where maternity/paternity was correct, were assumed to be known via DNA testing and so were *P*_*parent*_ *(AA)* = 1, *P*_*parent*_ *(Aa)* = 0, *P*_*parent*_ *(aa)* = 0. Sire genotype probabilities where paternity was incorrect were taken as the genotype frequencies within the background (untested) population assumed to remain in Hardy-Weinberg equilibrium, with the recessive disease causing mutant allele at a set frequency, and so constant. The resultant genotype frequencies for progeny assigned HC status were calculated (*P*_*progeny_n*_*(AA), P*_*progeny_n*_*(Aa), P*_*progeny_n*_*(aa)*), and these formed the sire and dam genotype probabilities (*P*_*parent_n + 1*_*(AA)*, *P*_*parent_n + 1*_*(Aa)*, *P*_*parent_n + 1*_*(aa)*), when maternity/paternity was correctly recorded) for the next generation. Calculations were performed iteratively over 10 generations of HC status assignment from when genotypes were ascertained by DNA testing, over a range of disease causing mutation frequencies (*f(a)* = 0.01, 0.05, 0.1, 0.2 and 0.3), using R [15]. The details of the probability calculations are given in the Appendix. The FHCR, or probability that the HC status assigned to an individual was incorrect, per generation, was calculated as 1 – P_*progeny*_ (AA).

**Fig. 2:**
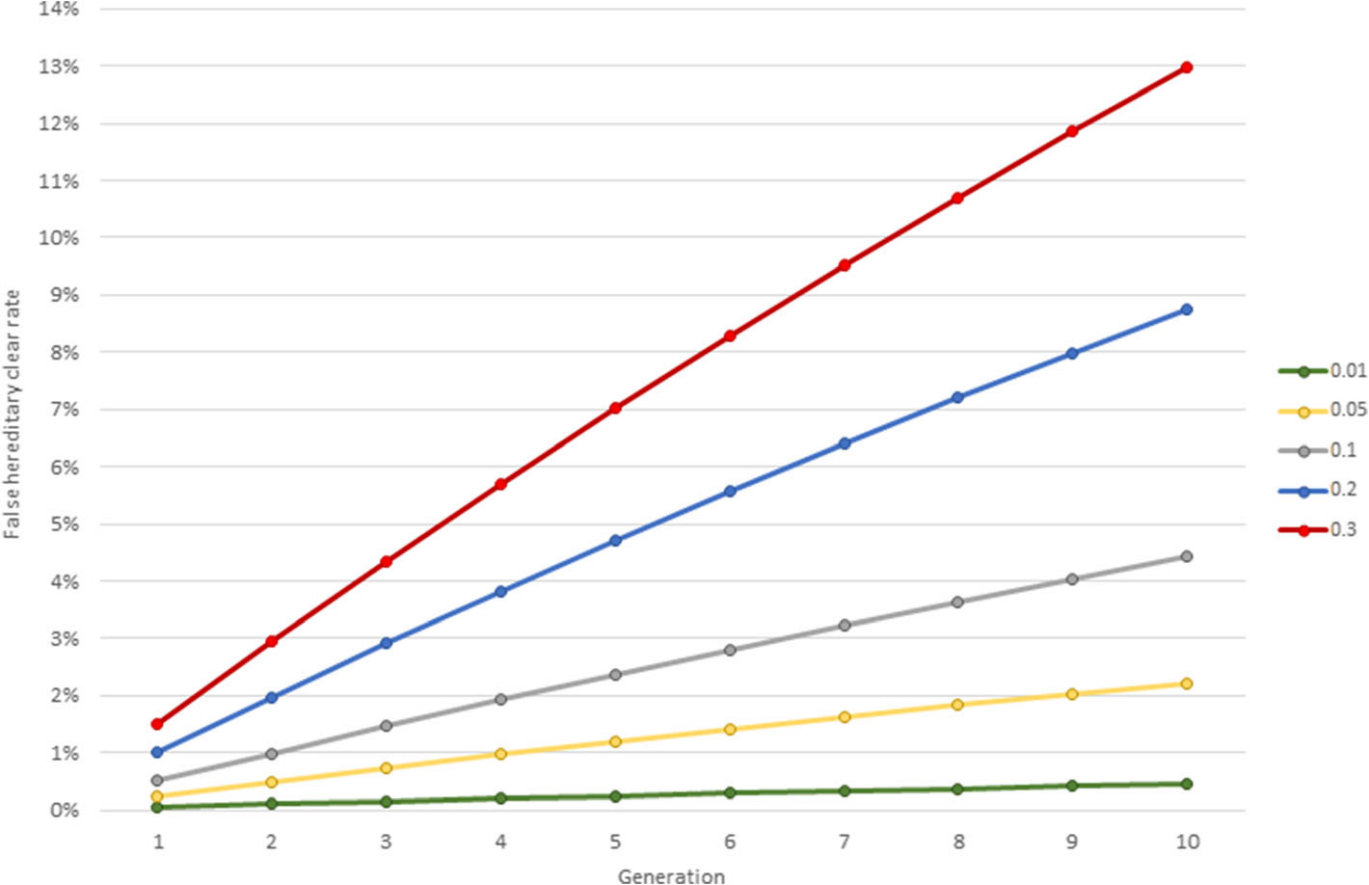
False hereditary clear rate over 1 to 10 generations of assignment at disease causing mutation frequencies 0.01, 0.05, 0.1, 0.2 and 0.3

The cumulative FHCR derived over 1 to 10 generations of HC assignment and at the various disease causing mutation frequencies are given in Table 1 and shown graphically in Fig. 2.

**Table 1:** The cumulative false hereditary clear rate (as a percentage) over 1 to 10 generations, at disease causing mutation frequencies 0.01, 0.05, 0.1, 0.2 and 0.3

generation	disease causing mutation frequency				
	0.01	0.05	0.1	0.2	0.3
1	0.05%	0.25%	0.50%	1.00%	1.50%
2	0.10%	0.49%	0.99%	1.97%	2.95%
3	0.15%	0.73%	1.46%	2.91%	4.35%
4	0.19%	0.96%	1.92%	3.82%	5.70%
5	0.24%	1.19%	2.36%	4.70%	7.01%
6	0.28%	1.40%	2.80%	5.56%	8.28%
7	0.32%	1.62%	3.22%	6.39%	9.51%
8	0.37%	1.83%	3.63%	7.20%	10.70%
9	0.41%	2.03%	4.03%	7.99%	11.86%
10	0.45%	2.22%	4.42%	8.75%	12.97%

The FHCR when HC status is first assigned to progeny of tested parents (i.e. generation = 1) is the product of the false paternity rate (0.05) and the disease causing mutation frequency (e.g. 0.05 × 0.1 = 0.005, or 0.5%; see Table 1). As the number of ancestral generations over which HC is assigned rises, the FHCR increases due to the cumulative probability of the nominated parents not having the AA genotype (despite HC status) due to false paternity in the intervening ancestral generations. In contrast, the false paternity rate and genotype frequencies in the background population (from which the probabilities of *actual* sire genotypes were sampled) were assumed constant per generation.

The cumulative FHCR rises quickly over early generations for disease causing mutations at a high frequency (0.2 and 0.3), exceeding 5% (1 in 20 erroneous) when HC status is assigned over 6 and 4 consecutive generations respectively (Table 1). However, even at the much lower disease causing mutation frequency of 0.05, a FHCR of over 2% is reached when HC status is assigned over 9 consecutive generations, implying that more than 1 in 50 individuals assigned HC status from 9 consecutive ancestral generations would not have the homozygous wild type (*AA*) genotype. Only when the disease causing mutation frequency was very low (0.01) did the FHCR not rise to a notable level over the course of 10 consecutive generations of HC status assignment (approximately 1 in 220; Fig. 2).

### Implications

The results presented here demonstrate that the rate of false HC status assignment can rise to considerable levels over a fairly small number of generations, simply due to erroneous recording of parentage. This rate is dependent on the frequency of the disease causing mutation in the population; when the mutation is relatively common (0.2 to 0.3), about 1 in 20 ‘hereditary clear’ individuals at 4–6 generations may been assigned so erroneously and in fact have either a heterozygote, or even mutant homozygote, genotype.

The implications of the results presented here are serious and deserve full consideration. Although where erroneous HC status occurs the error is often eventually discovered, this will almost always involve the production of affected individuals from two nominally ‘clear’, but actually ‘carrier’, parents, resulting in puppies destined to be affected by diseases which frequently have a hugely detrimental impact on welfare. Indeed, the *raison d’etre* of DNA testing for autosomal recessive disease causing mutations is to ensure that no affected individuals need ever be born again, while enabling breeders to select to bring the disease causing mutation frequency down to negligible levels without imposing a genetic bottleneck on the breed. Therefore, errors in HC status due to erroneous recording of parentage violate the principle objective of using DNA tests and have the potential to risk significant and unnecessary distress to both dogs and their owners.

The disease causing mutation frequencies over which FHCR was calculated in this study ranged from the very rare (0.01), where the incidence of disease in a generation produced by random mating would be just 1 in 10,000 (0.01%), to the really quite common (0.3), with a corresponding incidence of just less than 1 in 11 (9%). This range corresponded approximately to mutation frequencies reported by Lewis and Mellersh [13], which ranged from 0.0067 for Hereditary Cataract in the Staffordshire Bull Terrier to 0.28 for PRA-rcd4 in the Gordon Setter (frequencies calculated prior to DNA test availability). When the mutation frequency was very low (0.01) the FHCR remained relatively small, even over 10 generations of HC status assignment, at 0.45% or the HC status of 1 in ~ 220 individuals projected as being false. This is due to the comparative high frequency of the (non-disease causing) wild type allele (*A)*, meaning that when paternity was incorrect, the *actual* sire (assumed sampled at random with respect to genotype) was very likely to still have the AA genotype (with a probability of 0.98). Thus the resulting progeny were also very likely to inherit the AA genotype; the same genotype as implied by HC status, despite incorrect paternity. In contrast, where the disease causing mutation frequency is high, the probability of the *actual* sire still having the AA genotype is much lower; even being less than half (0.49) when the mutation frequency is 0.3, with the probability of 0.42 of him being an [undetected] carrier. It is this means via which the FHCR grows steadily over successive generations when mutation frequency is high, so that after 10 generations of HC status assignment more than 1 in 8 individuals are projected to have erroneous HC status.

The causes of incorrect parentage will likely include administrative errors in recording the identity of sire and/or dam in registration documentation and cases of ‘accidental’ matings unknown to the breeder/owner, as well as deliberate misinformation. However, the occurrence of incorrect parentage is all but inevitable and is documented in virtually all domesticated species, as well as humans [16]. The rate of incorrect paternity in this simulation is the middle of the range of pedigree error reported in domestic dog breeds by Leroy et al. [14].

This analysis makes several assumptions for the sake of brevity and simplicity which will not be true in real world populations. These include discrete (non-overlapping) generations, that the identity of one parent was only ever incorrectly recorded, and that the disease causing allele was initially in Hardy-Weinberg equilibrium in the population (and remained so in the proportion of the population without known genotypes, from which the ‘actual’ sire was drawn). In this simulation, it was assumed that that the population was homogeneous, with no ‘sub-structure’ (and so no differential mutation frequency across sub-populations). However, it is unlikely that the disease causing mutation frequency in dogs with unknown genotype would remain at the level described in the population at the outset, particularly at high frequencies when the prevalence of the disease would also be very high. In cases of false paternity, the ‘actual’ sire also being HC was not considered here, which if there is substructure in the breed and/or a strong uptake of testing delivering a considerable proportion of the breed with known genotypes may be a consideration. These assumptions may have led to an over-estimate of FHCR. Also not considered were HC to ‘carrier’ matings, which with DNA testing of the resultant litter may be used to safely incorporate carrier stock into breeding strategies and lessen the risk of a genetic bottleneck. Such matings are at increased risk of producing affected puppies when HC status is erroneous. Finally, error due to failure of laboratory protocols at testing was not considered. Nevertheless, this brief study succinctly demonstrates the problematic issue of perpetual assignment of HC status across generations.

It may be argued that the majority of dogs assigned HC status are destined to become pets, and so neutered, rendering false HC status irrelevant. While it is undoubtedly true that HC status may be applied to an entire litter, whereas genotypes ascertained by DNA tests apply only to the individual, breeders should nevertheless be aware of the issues of FHCR when HC is assigned over several generations and repeat tests every so often.

In light of the results from this simulation, the UK Kennel Club Board has considered that the assignment of HC status should be curtailed to reduce the risk that any errors in the recording of parentage result in the unintentional breeding of affected puppies. This proposal was discussed among the Kennel Club’s various health committees and a subsequent recommendation that HC status should be limited to 2 generations (with re-testing undertaken at a minimum of every 3 generations) from 1st January 2022 was agreed. This updated policy will therefore require a dog with parents and grandparents with HC status to undergo testing itself to confirm its genotype. Where parentage is confirmed by DNA profile, it is recognized that incorrectly recorded parentage, the major contributor to erroneous HC status, will be identifiable early (i.e. at the first generation) allowing the correction of publically accessible results before any affected puppies are born. Therefore, it has been agreed that, where parentage is confirmed by DNA profile, HC status may continue to be bestowed in perpetuity.

## Conclusion

The issue of incorrect recording of parentage has a clear potential impact on the inadvertent production of puppies affected by diseases for which DNA tests are widely available when HC status is assigned over several generations. The Kennel Club has taken the decision to curtail the number of generations over which HC status can be assigned, unless parentage is confirmed by DNA profiles.

## Data Availability

Data sharing is not applicable to this article as no datasets were generated or analysed during the current study.

## Appendix

Genotype frequencies were calculated at a hypothetical autosomal locus ‘A’, with a recessive disease causing mutant allele (*a*) occurring at frequency *f(a)*, in a population assumed to be in Hardy-Weinberg equilibrium:

$$ \mathrm{P}(AA)={\left(1-f(a)\right)}^2 $$

$$ \mathrm{P}(Aa)=2\left(\left(1-f(a)\right).f(a)\right) $$

1 $$ \mathrm{P}(aa)=f{(a)}^2 $$

‘Hereditary clear’ (HC) status is conferred on the progeny of two nominated parents both of whom are determined as having the homozygous wild type genotype (*AA*). A consistent rate of incorrect parentage, false paternity rate (*fpr)*, was considered in one parent [sire] only. The genotype probabilities where parentage/paternity is *incorrect* (*GP*_*false*_) is:

2 $$ {GP}_{false}=\mathrm{P}\left( genotypes\mid incorrectpaternity\right)= fpr\times \left[\begin{array}{c}P(AA)\\ {}P(Aa)\\ {}P(aa)\end{array}\right] $$

Since *fpr* and the genotype frequencies in [1] are constant over generations, so is *GP*_*false*_. The parental genotype probabilities where nominated parentage is correct (*GP*_*true*_), in the first generation (*n* = 1) of HC status assignment, come from genotypes determined by DNA testing, and so are:

3 $$ {GP}_{true}=\mathrm{P}\left( genotypes\mid parentagecorrect\right)=\left[\begin{array}{c}1\\ {}0\\ {}0\end{array}\right] $$

Dam genotype probabilities (*DGP*) are equal to *GP*_*true*_ since it was assumed that dam was recorded without error. Sire genotype probabilities (*SGP*) are calculated as:

4 $$ SGP=\left(\left(1- fpr\right).{GP}_{true}\right)+{GP}_{false} $$

A 3 × 3 ‘Punnett square’ matrix of genotypes (*M*) produced via mating is calculated:

5 $$ M= SGP.{DGP}^T $$

From which the vector of [progeny] genotype probabilities (*ProgG*) can be constructed:

6 $$ ProgG=\left[\begin{array}{c}P(AA)\\ {}P(Aa)\\ {}P(aa)\end{array}\right]=\left[\begin{array}{c}M\left[1,1\right]+\frac{1}{2}M\left[1,2\right]+\frac{1}{2}M\left[2,1\right]+\frac{1}{4}M\left[2,2\right]\\ {}\frac{1}{2}M\left[1,2\right]+\frac{1}{2}M\left[2,1\right]+M\left[1,3\right]+M\left[3,1\right]+\frac{1}{2}M\left[2,2\right]+\frac{1}{2}M\left[2,3\right]+\frac{1}{2}M\left[3,2\right]\\ {}\frac{1}{4}M\left[2,2\right]+\frac{1}{2}M\left[2,3\right]+\frac{1}{2}M\left[3,2\right]+M\left[3,3\right]\end{array}\right] $$

Over incremental generations (*n* = 2 → 10) of HC assignment, *GP*_*true*_ in [3] in generation *n* is equal to *ProgG* produced in generation *n-1* (i.e. progeny genotype probabilities become parental genotype probabilities in the next generation, where nominated parentage is true).

The false hereditary clear rate (FHCR), or probability that the HC status assigned to an individual was incorrect, per generation is calculated as:

7 $$ {\displaystyle \begin{array}{c} FHCR= ProgG\left(P(Aa)\right)+ ProgG\left(P(aa)\right)\\ {} or\\ {}=1- ProgG\left(P(AA)\right)\end{array}} $$

Data were generated for *n* = 1:10 generations of HC status being assigned after determination of genotypes from DNA testing in both parents, at a range of disease causing mutation frequencies (*f(a)* = 0.01, 0.05, 0.1, 0.2, and 0.3.), and with *fpr* = 0.05.
